# Continued misinterpretation of confidence intervals: response to Miller and Ulrich

**DOI:** 10.3758/s13423-015-0955-8

**Published:** 2015-11-30

**Authors:** Richard D. Morey, Rink Hoekstra, Jeffrey N. Rouder, Eric-Jan Wagenmakers

**Affiliations:** Cardiff University, Cardiff, UK; University of Groningen, Groningen, Netherlands; University of Missouri, Columbia, MO USA; University of Amsterdam, Amsterdam, Netherlands

**Keywords:** Statistics, Bayesian statistics, Statistical inference

## Abstract

Miller and Ulrich (2015) critique our claim (Hoekstra et al., *Psychonomic Bulletin & Review, 21*(5), 1157–1164, 2014), based on a survey given to researchers and students, of widespread misunderstanding of confidence intervals (CIs). They suggest that survey respondents may have interpreted the statements in the survey that we deemed incorrect in an idiosyncratic, but correct, way, thus calling into question the conclusion that the results indicate that respondents could not properly interpret CIs. Their alternative interpretations, while correct, cannot be deemed acceptable renderings of the questions in the survey due to the well-known reference class problem. Moreover, there is no support in the data for their contention that participants may have had their alternative interpretations in mind. Finally, their alternative interpretations are merely trivial restatements of the definition of a confidence interval, and have no implications for the location of a parameter.

Understanding how to interpret confidence intervals is critical to researchers. Particularly in the current climate of methodological introspection, it is essential that we understand methods in order to decide whether these methods are worthwhile. In our paper (Hoekstra et al., 2014, henceforth HMRW), we presented results from a survey with six statements about confidence intervals, all of which we claimed were false. These six false statements were endorsed at high rates, which we took as evidence that survey respondents had deep misunderstandings of confidence intervals.

Miller and Ulrich (2015, henceforth MU), however, argue that some of the statements we considered incorrect could be “appropriate under other meanings of ‘probability’ that are in common use” (Miller & Ulrich, 2015, p. XX); hence, they argue that our conclusion does not follow. In this response to MU, we address two related questions: first, are MU correct that some of the statements in HMRW could be considered correct by a mere reinterpretation of the wording? Second, to what extent do the data show responding consistent with MU’s interpretation? We will show that the reason why the statements are false is deeper than any interpretation of the word “probability”. Further, the data show strong evidence of confusion over confidence intervals, and no evidence that participants were responding as MU would predict, even if MU were correct about the interpretation of CIs.

It is worth emphasizing that the points we make about confidence intervals, and probability in general, are not controversial from the the point of view of statistical theory; they have been emphasized by frequentist and Bayesian philosophers and statisticians since the inception of CI theory in the 1930s, even by the authors cited by MU (Robinson, 1975, 1979; Dempster, 1964; de Groot & Schervish, 2012). The literature on this topic is vast and stretches back a century. In this response, we only just touch on this literature. In another article, we give a more in-depth treatment to the topic of confidence interval theory (Morey et al., 2015). We have intentionally chosen here to avoid repetition of the material in that article; readers will thus benefit from reading both articles.

## Can we be 95 % bunky?

MU are correct that much of the confusion about probability and confidence intervals is due to word choice. Both the word “probability” and the word “confidence” have meanings outside of their technical ones in frequentist statistics. In fact, commentators on CI theory have noted that this is part of the problem (Dempster, 1964; Mayo, 1981; Morey et al., 2015); users of CIs, for instance, incorrectly believe that “confidence” can be understood in its lay sense.

In their survey, HMRW offered a 95 % confidence interval for a mean of [.1,.4] — not giving any information about how it was derived — and then asked whether six statements followed from that information. For instance, statement 4 was:

### **Statement 4 (HMRW)**

*There is a 95 % probability that the true mean lies between 0.1 and 0.4*.

This statement does not follow from the information given under any common definition of probability, and so we took endorsement of this statement as indicating that participants misunderstood confidence intervals. HMRW briefly explained that statement 4 was one of several statements that “assign probabilities to parameters or hypotheses, something that is not allowed within the frequentist framework.” MU admit that “statement 4 is incompatible with a strict frequentist interpretation in which the current CI is regarded as a single isolated instance,” and give a brief review of commentators explaining that the frequentist probability that an observed interval contains the true value is either 0 or 1, but imply that a mere reinterpretation of the word “probability” suffices to make the statement acceptable.

MU’s review, however, does not explain clearly *why* frequentists made such proscriptions in the first place. They refer to “strict frequentists,” conjuring the image of an overbearing parent trying to keep their child from using vernacular. This gives a false impression that the disagreements between frequentists, Bayesians, and others are merely linguistic. They are not. Interpretation of confidence intervals is limited by a fundamental philosophical issue that is much deeper than a linguistic one.

We begin by reviewing the concept of a confidence procedure. A confidence procedure (CP) is a procedure that generates confidence intervals, and is said to have a confidence coefficient of *X%* if, in repeated sampling, *X%* of intervals would contain the true parameter value for all values of the true value (Neyman 1937). The idea of a confidence procedure is conceptually very clear. The confidence coefficient is a so-called “pre-data” measure of the uncertainty that we have in whether a sampled interval will contain the true value. All the disagreement comes after the data are observed and an interval is computed. How do we then interpret a 95 % confidence interval? Does it have a 95 % probability of containing the true value? Neyman (1937) says “Consider now the case when a sample...is already drawn and the [confidence interval] given... Can we say that in this particular case the probability of the true value of [the parameter] falling between [the limits] is equal to [ *X%*]? The answer is obviously in the negative” (p. 349). Neyman is saying that the confidence coefficient is not to be used as a “post-data” probability; that is, an assessment of the probability that the specific interval computed from the data, includes the true value.

MU would have us believe that Neyman’s negative answer is merely because of his “strict frequentist” definition of probability. In truth, the problem is deeper. Statisticians of the era were well aware of a basic fact: attempts to associate properties of individual events with long-run frequencies suffer from what is called the *reference class problem* (Reichenbach, 1949; Venn, 1888; von Mises, 1957). To understand the reference class problem, consider the question of assessing the probability that a particular African-American women, Jane, will die of cancer. All frequencies consist of a numerator and a denominator: the numerator gives the number of events consistent with the property in question — in this case, dying of cancer — and the denominator gives the “reference class” of events that could have had that property. To determine the probability that Jane will die of cancer we need a reference class. We might choose as a reference class “women”, in which case we would accept the long-run relative frequency with which women die of cancer as our probability. Or, we might choose “African-Americans” as our reference class. Either one can be chosen as the probability that *Jane herself* will die of cancer, but which one? The two reference classes will lead to different probabilities.

We might then attempt to resolve the contradiction by looking at the long-run frequency with which African-American women die of cancer. This will yield yet a third probability, different from the first two. But Jane is not just an African-American woman; she also has a particular profession, lives in a particular nation, province, city, and neighborhood. All these reference classes, and their combinations, will yield a different probability. The most specific reference class contains only Jane herself, and she will either die of cancer or not.

The above example, adapted from Venn (1888), makes clear the broad implications of the reference class problem. As Pinker (1999) said, problems such as these “are not academic; they affect every decision we make” (p. 349). The reference class problem is the reason why frequentists do not associate long-run uncertainties with individual events. The question always arises, “which long run?”

With confidence intervals, as with Jane, there are many long runs to choose from. For any given estimation problem, there will be many methods of constructing a confidence interval. These methods will yield different intervals with the same confidence coefficient (e.g., Morey et al., 2015; Neyman, 1952). Likewise, one can obtain the same interval via multiple confidence procedures, each with different confidence coefficients (e.g., Pearson, 1939). The multiplicity of ways that “confidence” can be associated with the same interval causes a reference class problem. We will show, for instance, dividing normal-theory-based confidence intervals into “long” and “short” intervals based on an arbitrarily-chosen width criterion will give us new reference classes: long intervals will contain the true value more than 95 % of the time, and short ones less.

To show that the problem is not about the meaning of word “probability” as MU claim, we will do something that may seem strange: we will use the nonword “bunky” instead, associating it with certain kinds of intervals. We may say, for instance, that we have 95 % “bunkiness” in an interval. Note that bunky has no meaning at all, and yet we will show logical inconsistencies. Suppose we have a random sequence of confidence intervals generated from a confidence procedure, and we know that in the long run *X%* of them would contain the true value, then for any one of those intervals we say the interval is *X%* bunky, or that we have *X %* bunkiness in the interval. Put another way, we have *associated X* % bunkiness with the interval.

To show that “bunkiness” leads to problems in spite of its meaninglessness, we consider the case where we have a specific confidence interval to interpret. Suppose, for instance, we sample *N* = 10 participants from a normal population. Our sample mean is $\bar {X}=0.25$, and our sample standard deviation is *s* = 0.21, yielding a 95 % Student’s *t* confidence interval of [0.1,0.4]. We now say we have 95 % bunkiness in that interval.

Suppose that someone reliable tells us that *σ* = 0.15. We now have more information that we could use in the data analysis. Notice, however, that nothing about the long-run behaviour of the Student’s *t* intervals changes, so our bunkiness doesn’t change. Still, 95 % of the Student’s *t* intervals, on average, contain the true value; still, either the true value is in the interval or it is not, and the conditions under which the interval contains the true value have not changed. We thus still have 95 % bunkiness in the interval. But now, we could compute a 95 % *z* interval of [0.16,0.34]; another interval, nested within the first, in which we can have 95 % bunkiness. This is not yet a logical contradiction, but it does appear that bunkiness is a strange construct.

**Fig. 1:**
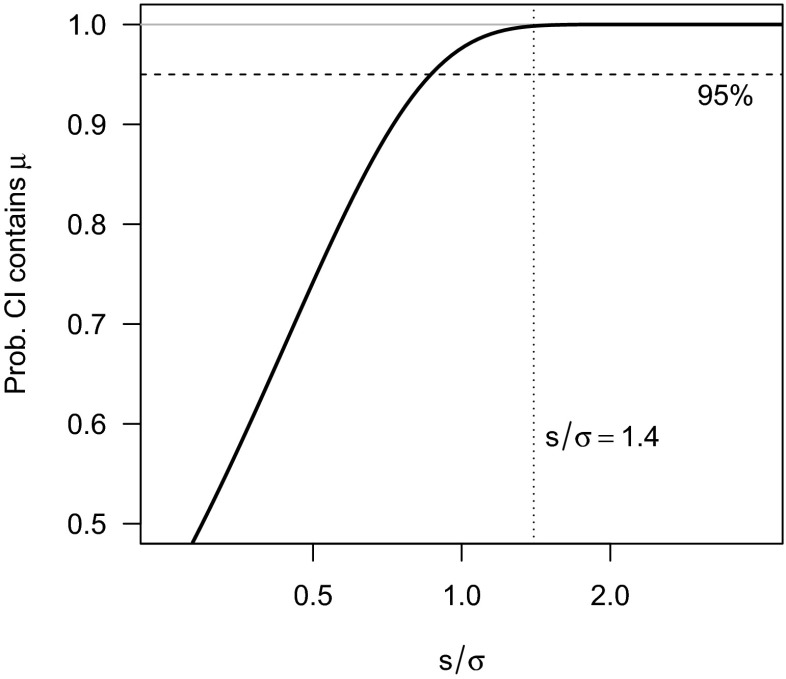
Probability that a 95 % Student’s *t*_9_ CI contains the true value as a function of the ratio of the sample standard deviation *s* to the true standard deviation *σ*

We can also obtain a second assessment of the bunkiness of our first interval. It turns out that 99.8 % of Student’s *t* intervals contain the true value when *s*/*σ* = 1.4, and thus we can have 99.8 % bunkiness in the interval [0.1,0.4]. This is due to the easily-proven but not widely-known fact that wider Student’s *t* CIs contain the true value with greater probability than narrower ones. Figure 1 shows the probability that an interval contains the true value as a function of the overestimation of the true standard deviation. Intervals are narrow when *σ* is underestimated, and wide when *σ* is overestimated.

We have now assigned both 95 and 99.8 % bunkiness to the same interval. This is the essence of the reference class problem. When the reference class is all intervals, the probability is .95; when the reference class is all intervals with *s*/*σ* = 1.4, the probability is 0.998. In statistics, the set of all samples such that *s*/*σ* = 1.4 is called a *relevant subset*, because knowledge that our sample is in this subset can inform our inference.

One might object that the above demonstration required knowledge of *σ*, but this does not diminish the force of the argument. The long-run properties of Student’s *t* remain exactly the same after knowledge of *σ* was obtained, and those are the properties on which the bunkiness was based. It is true that when *σ* is known the *z* interval is better, in the sense that the *z* interval will have a shorter average width; MU’s logic, however, did not include anything about optimality; in fact, they say that “[such] criteria have no relevance to the present discussion” (footnote 1, page XX). As it turns out, however, the objection is moot; we do not need to know the true value of *σ* to show a contradiction.

Examination of Fig. 1 reveals that the probability of an interval containing the true value is an increasing function of *s*. This suggests a strategy: pick any positive number *s*_0_, and if *s*>*s*_0_ call the interval “wide.” If *s*≤*s*_0_, call the interval “narrow.” A simple calculation will show that narrow intervals contain the true value less than 95 % of the time, while wide intervals will contain the true value more than 95 % of the time, regardless of what number we choose as *s*_0_. Suppose we chose *s*_0_ = .1. Since *s*>*s*_0_, we would call [.1,.4] a wide interval. We know that wide intervals contain the true value more than 95% of the time, and our interval is a wide interval; thus, we can have *more than* 95 % bunkiness in the interval.

We have now associated exactly 95 % bunkiness and more than than 95 % bunkiness with the same interval, which is clearly a contradiction. Showing this contradiction did not depend on the word “bunkiness” having any particular definition; rather, it depended only on the association between the “bunkiness” of the interval and a long-run probability. The issue cannot be defined away; simply replace the word “bunkiness” with “confidence” or “probability,” and the problem is the same.^1^

This problem with confidence intervals has long been understood by statisticians. Fisher, for instance, understood the problem immediately. In the first published discussion of confidence interval theory, Fisher stated that confidence interval theory “had been erected at considerable expense, and it was perhaps as well to count the cost. The first item to which [Fisher] would call attention was the loss of uniqueness in the result, and the consequent danger of apparently contradictory inference.” (Neyman, 1934, discussion at p. 618). Fisher’s critique of confidence intervals did not arise because he was reluctant to associate probabilities with intervals; Fisher had developed fiducial theory, which allows precisely that. Fisher’s critique of CI theory arose because CI theory does not afford a unique association between the probabilities and intervals. Neyman and Pearson were both aware of this fact. After giving an example wherein the same interval could be given two different probability assignments, Pearson (1939) wrote:

“Following Neyman’s approach [of not associating a probability to specific intervals], there is no inconsistency in this result, since one probability is associated with the employment of the [one procedure], the other with [another procedure]. It is only when we try to divorce the probability measure from the rule and to regard the former as something associated with a particular interval, that the need for a unique probability measure seems to be felt.” (p. 471)

The way that Neyman avoided inconsistency was to avoid, as Pearson put it, divorcing probability statements from the procedures: “What [the CI] assert[s] is that the [long-run] probability of success in estimation [...] is equal to [ 95 *%*]” (Neyman, 1952). Neyman denied that confidence intervals provided *any conclusions* about the parameter:

“[I]t is not suggested that we can ‘conclude’ that [the interval contains *μ*], nor that we should ‘believe’ that [the interval contains *μ*]...[we] decide to behave as if we actually knew that the true value [is in the interval]. This is done as a result of our decision and has nothing to do with ‘reasoning’ or ‘conclusion’. The reasoning ended when the [CI procedure was derived]. The above process [of using CIs] is also devoid of any ‘belief’ concerning the value [...] of [ *μ*].” (1941, p. 133–134)

To modern readers this will seem extreme, but it is actually a necessary restriction on the interpretation of confidence intervals, and attempts to deny it will fall victim to the reference class problem.

We can now see why the distinction between pre-data and post-data assessments of uncertainty is so critical in frequentism, and why the association between a probability (or confidence) statement and a specific interval in HMRW’s statements 1–5 were incorrect. It is not due to a linguistic prohibition by “strict frequentists”; rather, it is due to the reference class problem. Before observing the data, the reference class for “What is the probability that an interval in this sequence of intervals contains the true value?” is clear, because the reference class is defined by the procedure. However, “What probability/confidence should be associated with this specific interval?” is a question that cannot be uniquely answered based solely on the frequentist properties of a confidence procedure, because the interval will be a member of multiple reference classes. Any unique, post-data inference must be based on something other than freqentist long-run probability.

For more on relevant subsets and confidence intervals, see Buehler (1959), Buehler and Feddersen (1963), Brown (1967), Robinson (1975), Robinson (1979), and our in-depth discussion of confidence interval theory (Morey et al., 2015).

## Can experts be 95 % confident?

MU cite several authors who present statements about confidence intervals which we deem incorrect. MU are right that many papers and textbooks show interpretations of CIs which we would consider incorrect, and some are indeed written by well-known statisticians. Given the previous demonstrations that *p* values are often misinterpreted (e.g., Haller & Krauss, 2002), it should come as no surprise that CIs are also misinterpreted. It appears to be common knowledge among theoreticians that even experts can find confidence intervals difficult to understand.

MU assert that “it is implausible that such well-established mathematical statisticians do not understand CIs.” There are, however, several reasons why texts might contain incorrect or misleading statements about CIs. We explore two here: first, confidence interval theory is, indeed difficult, as indicated by the continuing controversy over how to interpret them. Second, writers of textbooks have different pressures on them than survey-takers; they want to write in a way that seems clear, and that connects with what people will read and hear elsewhere.

Even the literature cited by MU underscores that mathematical statisticians have difficulty with CIs. Dempster (1964) writes that “*[i]t does not appear to be widely understood that, after the observation is taken, the defining property [of the confidence interval] admits only a postdictive interpretation* [that is, the one suggested by MU].” (p. 57; emphasis in original). Dempster, in fact, emphasizes this sentence for effect. He is talking about mathematical statisticians, and explicitly saying that they do not appear to understand confidence intervals.

Neyman (1952, pp. 211–215) presents a humorous, fictional dialogue in which an “eminent elderly statistician” has great difficulty understanding confidence intervals. Mayo (1981) writes that “while confidence levels are often (wrongly) interpreted as providing [...] a measure of [certainty that the parameter is in the interval], no such interpretation is warranted. Admittedly, such a misinterpretation is encouraged by the word ‘confidence’.”

More recently, Briggs (2012b) described the definition of confidence intervals as “so contrived and anti-intuitive” that “[e]ven trained statisticians, who should know better, err and treat the confidence interval as a [Bayesian] credible interval” (p. 3–4). Elsewhere, Briggs (2012a) offered a humorous challenge: “If you can find even one [published analysis] where the confidence interval is not interpreted as a credible interval, then I will eat your hat.” None of the statisticians or philosophers quoted here take it for granted that statisticians understand confidence intervals; we see no reason why MU should take it for granted either.

It will be informative, however, to take a closer look at the three quotes from statistical experts that MU provided. Three textbooks written by experts used language close to our statement 5:

### Statement 5 (HMRW)

*We can be 95 % confident that the true mean lies between 0.1 and 0.4*.which we asserted was incorrect because it confuses the pre-data confidence coefficient with the post-data interval, and will thus will fall victim to the reference class problem.

Consider first de Groot (1989), which MU quote in its second edition: “[w]e can then make the statement that the unknown value of *μ* lies in the interval [...] with confidence 0.95.” This text is now in its fourth edition (de Groot and Schervish, 2012); the current version of the text *does not contain* the passage MU emphasize. The text later makes clear why it was removed, emphasizing that “the observed interval...is not so easy to interpret...[S]ome people would like to interpret the interval...as meaning that we are 95 % confident that *μ* is between [the observed confidence limits]. Later...we shall show why such an interpretation is not safe in general” (p. 487). Following an example (coincidentally, one explored by Morey et al. 2015) and dismissed by MU as an “artificial case”), the reason is given:

“it is not always safe to assume that our estimate is close to the parameter just because the sampling distribution of the estimator had high probability of being close. There may be other information available that suggests to us that the estimate is not as close as the sampling distribution suggests, or that it is closer than the sampling distribution suggests.” (p. 493)

As often happens in new editions of a text, the mistakes of the old edition have been corrected in the new. The new edition clearly states that the language used in the old edition cited by MU was generally incorrect and misleading.

Some authors make the friction between correctness and pragmatics explicit. Howell (2013), for example, states that “So what does it mean to say that the 95 % confidence interval is 1,219≤*μ*≤1,707? For seven editions of each of two books I have worried and fussed about this question” (p. 194). Hoenig and Heisey (2001) suggest that less-than-rigorous teaching of confidence intervals might even be acceptable, saying that “[i]f informally motivated confidence intervals lead to better science than rigorously motivated hypothesis testing, then perhaps the rigor normally presented to students destined to be applied researchers can be sacrificed” (p. 23). It must be emphasized, however, that an argument that a technically-incorrect statement might lead to better practical outcomes, even if true, is not an argument for the statement’s correctness.

A second reason why textbooks may contain incorrect statements is that the author is trying to connect with the conventions that have arisen around confidence intervals. The third example cited by MU was Robinson (1982), who said that “...we say that we have 95 % confidence...” (p. 121). Robinson, however, is well-known for his articles showing that the interpretation of confidence intervals is problematic. Indeed, in the opening of the entry quoted by MU, Robinson employs trademark British understatement in noting the problem:

“It is natural to expect it to mean ‘an interval in which one may be confident that a parameter lies.’ Its precise technical meaning *differs substantially from this* (see Jones 1958; Cox 1958, and Dempster, 1964) but the intuitive idea is not entirely misleading.” (p. 120, emphasis added)

Later, Robinson says that “confidence coefficients are a good measure of uncertainty before the data have been seen, but may not be afterward” (p. 125). Other papers by Robinson (1975) and (1979) — in which he calls such statements “unreasonable” and “rather dubious” — make the reasons clear: relevant subsets, as we described above. By using the offending language Robinson meant only to describe a convention, one that he explains is not correct in general. MU suggest that

“...saying that we are ‘95 % confident’ of a statement appears to be just a compact way of saying, ‘the statement is a random selection from a population of statements that are known to be 95 % accurate overall, and we have no other basis on which to judge the accuracy of the statement.’ ” (p. XX)

MU are incorrect, even according to the sources that MU cited for support: the reference class problem, and pervasive relevant subsets, tells us that we *do* have other bases on which to judge the accuracy of the statement, if the “accuracy” is the frequentist long-run probability.

A close examination of two of the three examples produced by MU therefore support the position taken by HMRW, and the third is likely a pragmatic oversimplification. We suspect readers will sympathize; what author has not, at some point, regretted oversimplifying a complicated concept to the point where it is incorrect? It does not require a stretch of the imagination to see why authors of texts might use incorrect language in describing confidence intervals, even without resorting to the explanation that the authors did not understand confidence intervals. Ultimately, however, what is correct and incorrect must be determined by theory, not by our impression of the status, knowledge, or motivations of textbook writers.

## How can one interpret an observed confidence interval?

Given that we have ruled out all of the common ways of interpreting confidence intervals, the question naturally arises: how can a single observed interval be interpreted in terms of the parameter of interest? There are, broadly speaking, three options. The first is to follow Neyman and avoid the problem by *not* interpreting CIs at all. As Neyman said, the confidence interval asserts nothing except that it is a sample from a confidence procedure.

Avoiding any interpretation is unacceptable for most scientists; after all, the goal of most research is often to make data-informed statements about the parameter. The second option is to interpret the interval as all the values that would not be rejected by a particular significance test. This, however, inherits all the problems of significance tests (Berger and Sellke, 1987; Jeffreys, 1961; Rouder et al., 2009; Wagenmakers et al., 2008). It is a well-known fact that failure to reject a value by a significance test cannot be used to argue that the value is reasonable, so we have merely pushed the interpretation problem back one level onto the significance tests, which is troubling. Moreover, this route violates the purpose that many researchers see confidence intervals as serving: to wean researchers off significance tests (Cumming, 2014; Loftus, 1996; Steiger, 2004; Steiger & Fouladi, 1997, e.g.).

The third route is to abandon confidence interval theory, and adopt another theory of inference. For instance, under certain conditions — namely given the specification of a prior distribution — Bayesian theory allows the assessment of the “posterior probability” of an interval, which indexes the post-data certainty with which an analyst should believe an interval contains the true value of the parameter. If a prior distribution is assumed, the intervals generated by confidence interval theory can be assessed for their posterior probability. The reason why this solution works is that the inference is not based on the long-run properties of the procedure, but rather the posterior distribution, and hence the inference must be unique. In some cases, Bayesian assessments of uncertainty will be similar to those obtained from a misunderstanding of confidence intervals. The Bayesian post-data probability, however, is guaranteed to be unique and meaningful, provided that the prior is meaningful.

MU offer other alternative interpretations of confidence intervals, which they claim are “appropriate” and “useful.”^2^ For instance, suppose one sampled data from a normal population and then computed a Student’s *t* interval of [.1,.4], and denote the standard error of the mean as *S*. They offer this statement as an alternative to HMRW’s statement 4:

### **Statement 4′ (MU)**

*If the current sample is one of the 95 % of all samples with relatively small values of*$|\bar {X}-\mu |/S$*, then μ lies in the interval 0.1-0.4.*

While true, this interpretation is trivial and unhelpful. To see why, let “relatively small” mean that $|\bar {X}-\mu |/S<c$ for some positive value *c*. Then a bit of algebra yields the condition

$$\bar{X} - cS < \mu < \bar{X} + cS. $$

But *c*, by definition, must be chosen such that 95 % confidence procedure results. This condition, then, is merely the condition that *μ* is in the interval. MU’s statement 4^′^ amounts to

### **Statement 4′ (reworded)**

*If the conditions under which μ would be in the interval hold, then μ lies in the interval; these conditions will hold in 95 % of samples*.

The first part of the statement is tautological; the only non-trivial information left is “The conditions under which *μ* is in the interval will hold in 95 % of samples.” Of course, this is merely the definition of the confidence procedure, and makes reference only to the long-run property of the interval. Although MU sought an alternative interpretation to the frequentist one, they have offered the frequentist definition in disguise.

The fact that MU’s definition turns out to assert only the long-run probability of the procedure would, of course, not surprise Neyman (1952), who specified that “[all the CI] does assert is that the probability of success in estimation ... is equal to [95 %]” (p. 214). It would also not surprise Dempster (1964), who said that such interpretations have “essentially tautological content” (p. 62). MU’s alternative statements are not useful; rather, they simply reassert that an interval is a confidence interval. MU’s statements give the researcher no new information and leave them with the same problem they had previously: wondering how to interpret the interval.

In fact, like all frequentist interpretations, MU’s statements do not have any implications for where one should believe the parameter actually is. Dempster points out that researchers actually desire a *different* sort of statement than the ones MU suggest: so-called “predictive” statements, ones that have implications for where the parameter is believed to be. He says “I find Neyman quite vague on the intellectual mechanism whereby [CI] interpretations come to have predictive implications. I suspect that particular observed confidence statements are intended to have the effect of predictive probability statements, without actually using the word probability and without paying the price which the use of the word probability demands.” (p. 60).^3^ We completely agree; the advocacy of confidence intervals rests on an interpretive sleight of hand by which trivial statements are rendered as predictive statements about the parameter (see also Morey et al., 2015).^4^

## How do participants interpret CIs?

A key aspect of MU’s argument is that “[the survey statements] are incompatible with a ‘strict frequentist’ interpretation of the word ‘probability’, but participants may have had a different interpretation in mind when completing the questionnaire. In that case, acceptance of these statements as true may not necessarily indicate misunderstanding of CIs per se, but rather use of a different interpretation of ‘probability’ ” (Miller & Ulrich, in press, p. XX). Although we reject their alternative interpretations, we can take another look at the data to see whether it is consistent with their view on how survey respondents may have been interpreting the questions.

Of course, we admit that interpretation of survey responses can be difficult. We cannot rule out that respondents may have interpreted our statements in any number of ways that we did not intend. In the case of our survey, the instructions and statements were short and simple. We believe the burden of proof lies with anyone suggesting that participants interpreted the statements to mean something other than what was written. As it turns out, we can easily show that participants *did not* have MU’s interpretation in mind, and even more, that the response patterns do not make sense under any definition of probability. We note that MU themselves admitted that our hypothesis — that respondents show robust misinterpretations of CIs — was “certainly support[ed].” Our goal in this section is to show that the case is stronger even than reported by HMRW, and inconsistent with MU’s proposed interpretation.

**Fig. 2:**
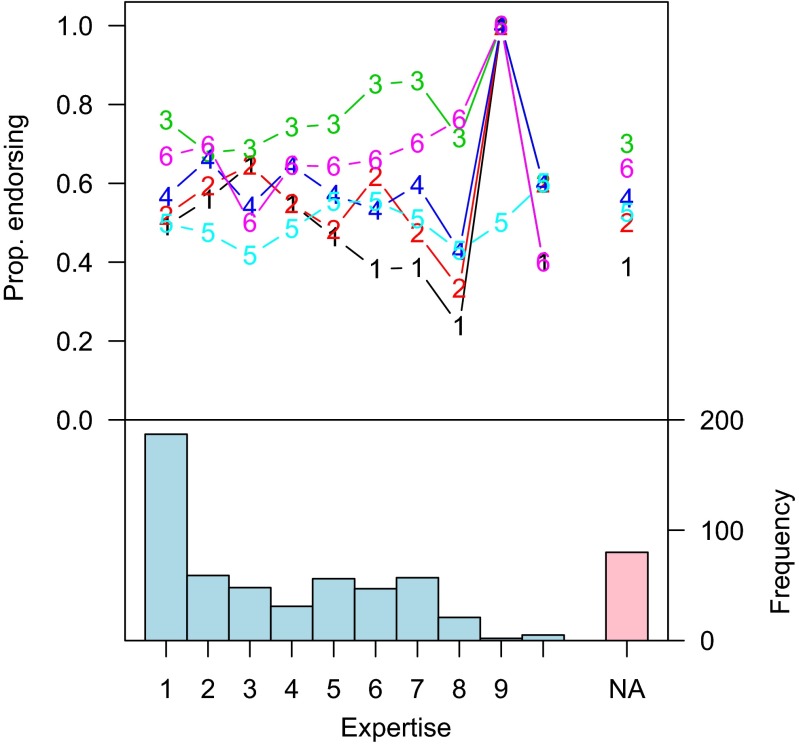
*Top*: Proportion of sample endorsing each statement as a function of self-rated expertise. Each series 1–6 represents the corresponding statement. If expertise is NA, then the respondent did not respond to this question. The vast majority of missing expertise ratings is from first-year students (86 %) or Master students (9 %). *Bottom*: The distribution of self-rated statistical expertise in the sample

Figure 2 (top) shows the proportion of endorsements for each statement as a function of self-rated expertise. Apart from statement 1, which shows a slight decrease in endorsements as expertise increases, there does not appear to be any relationship between expertise and endorsement of the statements. Consider statement 6 in HMRW:

### **Statement 6 (HMRW)**

*If we were to repeat the experiment over and over, then 95 % of the time the true mean falls between 0.1 and 0.4*.

This statement is nonsense, though the first author of this manuscript has heard it offered several times as an interpretation of confidence intervals. Statement 6, which should be easily rejectable by anyone with an understanding of CIs, has a higher endorsement rate than statements 4 and 5, endorsements of which MU claim may indicate knowledge of CIs. Moreover, endorsements of statements 4 and 5 do not increase as self-rated expertise increases. If CIs are difficult to interpret — as we assert and as MU admit — then it seems implausible that the responses of first-year students reflect no knowledge, while the equally-high endorsement rates of experienced researchers reflect knowledge. We believe it is much more plausible that these responses indicate lack of understanding.

Evidence that participants have a shaky understanding of confidence intervals can be found by examining the sheer diversity in the response patterns. Note that there are a total of 64 total possible response patterns for 6 true/false questions. Of these, 37 appear in the data for the non-students (*N* = 151). The most endorsed pattern by non-students, endorsed by 11 % of the non-students, is that all statements are correct. Note that this includes endorsement of nonsense statement 6. Interestingly, endorsing statement 6 alone is the next most popular response pattern (9 %), followed by statements 3 and 6 only (7 %). The top eight patterns are shown Table1

**Table 1:** The top 8 response patterns for non-students, and the proportion of non-students responding with that pattern

	Proportion
S1,S2,S3,S4,S5,S6	0.11
S6	0.09
S3,S6	0.07
S1,S2,S3,S4	0.05
S2,S3,S4,S6	0.05
S3,S4,S5	0.05
S3,S4,S6	0.05
*S1,S2,S3,S4,S5	0.05

The pattern predicted from MU’s re-interpretations is shown with an asterisk

MU suggest five alternative statements that could be endorsed under their interpretation of the survey, meant to replace our statements 1–5. If participants understood CIs in the way that MU suggest, then they would endorse statements 1–5, and reject statement 6. This response pattern was shown in only seven of the 151 non-student respondents (5 %).

Consider the seven response patterns that have at least as much endorsement as MU’s predicted pattern. Of these, five include endorsements of statement 6. Five do *not* include statement 5. MU argue that “HMRW’s participants may have interpreted statement 5 in the same way as the mathematical statisticians [and hence endorse it].” In the sample, 63 % of non-students endorsed nonsensical statement 6 that everyone — including mathematical statisticians — should reject, compared to 53 % who accept statement 5. Moreover, rejecting statement 6 was only mildly predictive of accepting statement 5; non-student respondents who rejected statement 6 accepted statement 5 61 % of the time, while non-student respondents who accepted statement 6 accepted statement 5 half of the time (correlation *ϕ* = −.11). Accepting statement 4 was only mildly predictive of accepting statement 5 (correlation *ϕ* =.18) even though, on MU’s analysis, the two statements have the same content. There does not appear to be evidence of any consistent interpretation in the data, including the one MU suggest.

Overall, an analysis of the data reveals little evidence that the survey respondents were interpreting CIs in the way MU suggest they might have been. MU offer no empirical evidence to support their contentions about what the participants might be thinking. The only evidence they offer in their response to HMRW is thought experiments with coins and cards. They imply that readers’ endorsement of their interpretation of these thought experiment supports their contention that people use their interpretation of probability; however, their thought experiments can be easily understood using Bayesian probability, and are hence not diagnostic.

The data do, however, offer more evidence that participants’ interpretations of confidence intervals reflect a lack of knowledge. Consider the necessary relationship between statements 1 and 4. Statement 1 was:

### **Statement 1 (HMRW)**

*The probability that the true mean is greater than 0 is at least 95 %*.and statement 4 was:

### **Statement 4 (HMRW)**

*There is a 95 % probability that the true mean lies between 0.1 and 0.4*.

Both statements make use of the word “probability”. Regardless of the interpretation of the word probability, the *laws* of probability must be respected. Assuming that respondents are interpreting the word probability consistently across questions, an endorsement of statement 4 implies that one must endorse statement 1: if the probability that the true mean is in [.1,.4] is 95 %, then there is *at least* a 95 % probability that the true mean is greater than 0, since all values in the CI are greater than 0. However, of non-students who endorsed statement 4, only 51 % endorsed statement 1. The responses are demonstrably internally inconsistent. In sum, responses patterns are highly varied, and often violate the laws of probability, even among experienced researchers. The most parsimonious explanation, by far, is that the survey responses indicate severe misunderstanding of CIs (and indeed, probability broadly).

One possible objection to the survey raised in review is that the survey contained no correct response, and hence might be seen as a “trick” survey. Respondents may have been confused, expecting there to be at least one right answer. In response, we note that only 8 % of respondents endorsed a single item, and a majority endorsed four or more items. We see no reason why confusion about there being no right answer would cause respondents to respond with more than one endorsement, much less four or more. Further, we note that CIs are considered a basic tool for researchers, taught from the first year of statistical training, used throughout the literature, and heavily promoted by statistical reformers as intuitive. If understanding of CIs is so fragile that merely leaving out a correct answer yields highly varied, internally inconsistent response patterns, this actually bolsters our case that CIs are badly misunderstood.

## Conclusion

MU’s arguments for asserting the appropriateness of the statements on HMRW’s survey do not hold. The reference class problem prevents any unique association of individual observed intervals with long-run frequencies. HMRW’s data provide ample evidence of misinterpretations of confidence intervals; responses were highly varied, uncorrelated with expertise, and often violate the laws of probability under any interpretation. Finally, the data are not consistent with how MU suggest participants may have been responding. The main conclusion of HMRW — that researchers have robust misunderstandings of confidence intervals — is sound. Confidence intervals are poorly understood not only by students and researchers, but also by methodologists such as MU who have given the matter considerable thought (see also Morey et al., 2015). This widespread lack of understanding, even among experts, should raise doubt about whether the widespread advocacy of confidence intervals is backed by a solid theoretical foundation.

## Footnotes

1 The problem is actually worse in the case of “probability,” because probability has to follow certain rules that neither “bunkiness” nor “confidence” do.

2 Though MU offer alternatives for our statements 1, 3 and 4, we will focus only on their alternative for statement 4; our points generalize to the other statements as well.

3 Compare this to MU’s statement that “the word ‘probability’ is conspicuously absent from [our] interpretations.”

4 Consider, for instance, Cumming’s (2014) statement that “a CI is usefully informative about what is likely to happen next time.” This is precisely what Dempster noted that confidence interval theory *isn’t informative about*.
